# lncRNA expression profiles and associated ceRNA network analyses in epicardial adipose tissue of patients with coronary artery disease

**DOI:** 10.1038/s41598-021-81038-5

**Published:** 2021-01-15

**Authors:** Qian-Chen Wang, Zhen-Yu Wang, Qian Xu, Xu-Liang Chen, Rui-Zheng Shi

**Affiliations:** 1 Department of Cardiovascular Medicine, Xiangya Hospital, Central South University, 87 Xiangya Road, Changsha, Hunan People’s Republic of China; 2 Department of Cardiovascular Medicine, The Second Xiangya Hospital, Central South University, Changsha, Hunan People’s Republic of China; 3 Department of Cardiovascular Surgery, Xiangya Hospital, Central South University, Changsha, Hunan People’s Republic of China

**Keywords:** Cardiovascular diseases, Cardiology

## Abstract

Epicardial adipose tissue (EAT) contributes to the pathophysiological process of coronary artery disease (CAD). The expression profiles of long non-coding RNAs (lncRNA) in EAT of patients with CAD have not been well characterized. We conducted high-throughput RNA sequencing to analyze the expression profiles of lncRNA in EAT of patients with CAD compared to patients without CAD. Gene Ontology (GO) and Kyoto Encyclopedia of Genes and Genomes (KEGG) pathway enrichment analyses were executed to investigate the principal functions of the significantly dysregulated mRNAs. We confirmed a dysregulated intergenic lncRNA (lincRNA) (LINC00968) by real-time quantitative PCR (RT-qPCR). Subsequently, we constructed a ceRNA network associated with LINC00968, which included 49 mRNAs. Compared with the control group, lncRNAs and genes of EAT in CAD were characterized as metabolic active and pro-inflammatory profiles. The sequencing analysis detected 2539 known and 1719 novel lncRNAs. Then, we depicted both lncRNA and gene signatures of EAT in CAD, featuring dysregulation of genes involved in metabolism, nuclear receptor transcriptional activity, antigen presentation, chemokine signaling, and inflammation. Finally, we identified a ceRNA network as candidate modulator in EAT and its potential role in CAD. We showed the expression profiles of specific EAT lncRNA and mRNA in CAD, and a selected non-coding associated ceRNA regulatory network, which taken together, may contribute to a better understanding of CAD mechanism and provide potential therapeutic targets.

*Trial registration* Chinese Clinical Trial Registry, No. ChiCTR1900024782.

## Introduction

Cardiovascular disease is the leading cause of death in the world. A large number of studies have confirmed that obesity is closely related to CAD. Recent studies have found in addition to the accumulation of peripheral fat, cardiac fat distribution is also a significant risk factor for cardiovascular disease^1,2^. EAT is visceral adipose tissue located between the heart and the visceral pericardium^3^. EAT is anatomically adjacent to the coronary arteries and myocardium, and directly contact with the coronary arteries without fascia separation. EAT can secrete a large amount of adipokines, affecting the physiological functions and pathophysiological processes of the myocardium and coronary arteries^4,5^. Various clinical studies have confirmed that EAT is closely related to the occurrence and development of coronary heart disease, but little research has been conducted on how EAT mediates the occurrence and development of CAD^6^.

LncRNA is a type of RNA with more than 200 nucleotides in length and lacks a complete open reading frame with little or no protein-coding ability^7^. lncRNA can regulate gene expression through different mechanisms of action, such as RNA-RNA base pairing, RNA–protein interaction, RNA–DNA interaction, etc.^8,9^. lncRNA has been reported to play an essential role in the regulation of cardiovascular physiological and pathophysiological processes such as heart development, heart failure, and CAD^10^. CeRNAs were described as groups of non-coding RNAs, mRNAs and other RNAs that compete with miRNAs at post-transcriptional level, by acting as molecular sponges for miRNAs via sharing miRNA response elements (MREs), thereby regulating mRNA expression and modulating downstream molecular processes^11^. ceRNA network links the function of protein-encoding mRNAs to the function of non-coding RNAs. In a target transcript, miRNAs that bind to MREs will attenuate the inhibitory activity of the miRNAs against other target genes. In theory, any transcript containing one or more MREs may play a role as a ceRNA. Therefore, the ceRNA regulation theory represents a widespread post-transcriptional regulation pattern of gene expression. An in-depth study of the regulatory mechanisms of ceRNAs can help reveal the pathogenesis of the disease.

At present, the expression and role of lncRNA in EAT of coronary heart disease has not been well characterized. In this study, we sought to explore the EAT lncRNA and mRNA expression profiles and look for a lncRNA-related ceRNA network and its role in disease pathogenesis of CAD and attempt to provide a new theoretical basis for targeted therapy of CAD.

## Results

### Study population

We enrolled 19 CAD patients who underwent coronary artery bypass graft surgery (CAD group), and 24 non-CAD patients who underwent cardiac valve replacement with negative clinical and evidence of CAD (CTRL group), of which 5 pairs of subjects’ EAT samples were used for high-throughput RNA Sequencing, the other samples were used for verifying the sequencing results. Compared with CTRL, CAD patients has higher proportion in hypertension and hyperlipidemia, while age, male sex, body mass index (BMI), abdominal circumference (AC), blood lipids, and left heart function showed no difference between two groups. Characteristics of the study population were provided in Table 1. We selected 10 subjects from the two groups matched in age, gender, BMI, abdominal circumference (AC), and RNA-seq were performed. The main clinical characteristics of those 10 subjects were summarized in Table 2. The other samples were used as an expansion for verification. Patients in both groups were routinely treated with heparin anticoagulation.

**Table 1:** Patients’ characteristics.

	CTRL (n = 24)	CAD (n = 19)	*p* values
Age (years)	57.0 ± 7.3	59.2 ± 8.8	0.364
Sex, male (%)	8 (32.0)	12 (68.0)	0.054
BMI (kg/m^2^)	23.7 ± 3.2	24.8 ± 2.4	0.241
AC (cm)	85.7 ± 10.2	89.2 ± 6.8	0.278
SBP (mmHg)	117.4 ± 12.1	124.8 ± 15.6	0.086
DBP (mmHg)	73.1 ± 12.9	71.9 ± 6.8	0.709
CAD			
1-vessel disease	0 (0.0)	2 (10.5)	
2-vessel disease	0 (0.0)	5 (26.3)	
3-vessel disease	0 (0.0)	12 (63.2)	
Complications			
Diabetes mellitus (%)	0 (0.0)	0 (0.0)	1.000
Hypertension (%)	5 (20.8)	10 (52.6)	0.032
Hyperlipidemia (%)	3 (12.5)	8 (42.1)	0.029
Laboratory measurements			
WBC (× 10^9^/L)	6.3 ± 2.3	6.2 ± 1.6	0.953
HbA1c (%)	2.7 ± 1.3	3.5 ± 2.3	0.448
TG (mmol/L)	1.4 ± 1.1	2.0 ± 1.2	0.085
LDL-c (mmol/L)	2.7 ± 0.6	2.7 ± 1.1	0.984
HDL-c (mmol/L)	1.1 ± 0.3	0.9 ± 0.2	0.070
Left heart function			
EF (%)	55.2 ± 8.8	57.1 ± 11.4	0.544
Medication			
β-blocker	10 (41.7)	17 (89.5)	0.001
ACEI/ARB	8 (33.3)	9 (47.4)	0.356
Statin	2 (8.3)	12 (63.2)	0.000

Data are presented as mean ± SD or the number (%) of patients.

*BMI* Body Mass Index, *AC* abdominal circumference, *SBP* systolic blood pressure, *DBP* diastolic blood pressure, *WBC* white blood cell, *HbA1c* glycated hemoglobin, *TGs* triglycerides, *LDL-c* low-density lipoprotein cholesterol, *HDL-c* high-density lipoprotein cholesterol, *EF* ejection fraction, *ACEI* angiotensin-converting enzyme inhibitors, *ARB* angiotensin receptor blockers.

**Table 2:** Clinical characteristics of 5 pairs subjects.

	CTRL (n = 5)	CAD (n = 5)	*p* values
Age (years)	57.4 ± 5.2	63.6 ± 11.5	0.305
Sex, male (%)	2 (40.0)	4 (80.0)	0.310
BMI (kg/m^2^)	21.4 ± 2.0	23.3 ± 2.5	0.223
AC (cm)	84.0 ± 8.9	91.2 ± 5.3	0.262
Diabetes mellitus (%)	0 (0.0)	0 (0.0)	1.000
CAD			
1-vessel disease	0 (0.0)	0 (0.0)	
2-vessel disease	0 (0.0)	2 (40.0)	
3-vessel disease	0 (0.0)	3 (60.0)	
Hypertension (%)	2 (40.0)	4 (80.0)	0.310
Hyperlipidemia (%)	0 (0.0)	1 (20.0)	0.317
LVEF (%)	54.8 ± 7.3	56.8 ± 13.6	0.780
Laboratory measurements			
WBC (× 10^9^/L)	4.4 ± 1.3	6.4 ± 2.3	0.147
HbA1c (%)	3.42 ± 1.94	3.41 ± 2.51	0.827
TG (mmol/L)	2.1(0.9–3.8)	1.9(1.2–3.1)	0.753
TC (mmol/L)	4.1 ± 1.1	3.8 ± 0.6	0.518
LDL-c (mmol/L)	2.6 ± 0.8	2.3 ± 0.5	0.548

### Identification of differentially expressed lncRNA and mRNA

**Figure 1:**
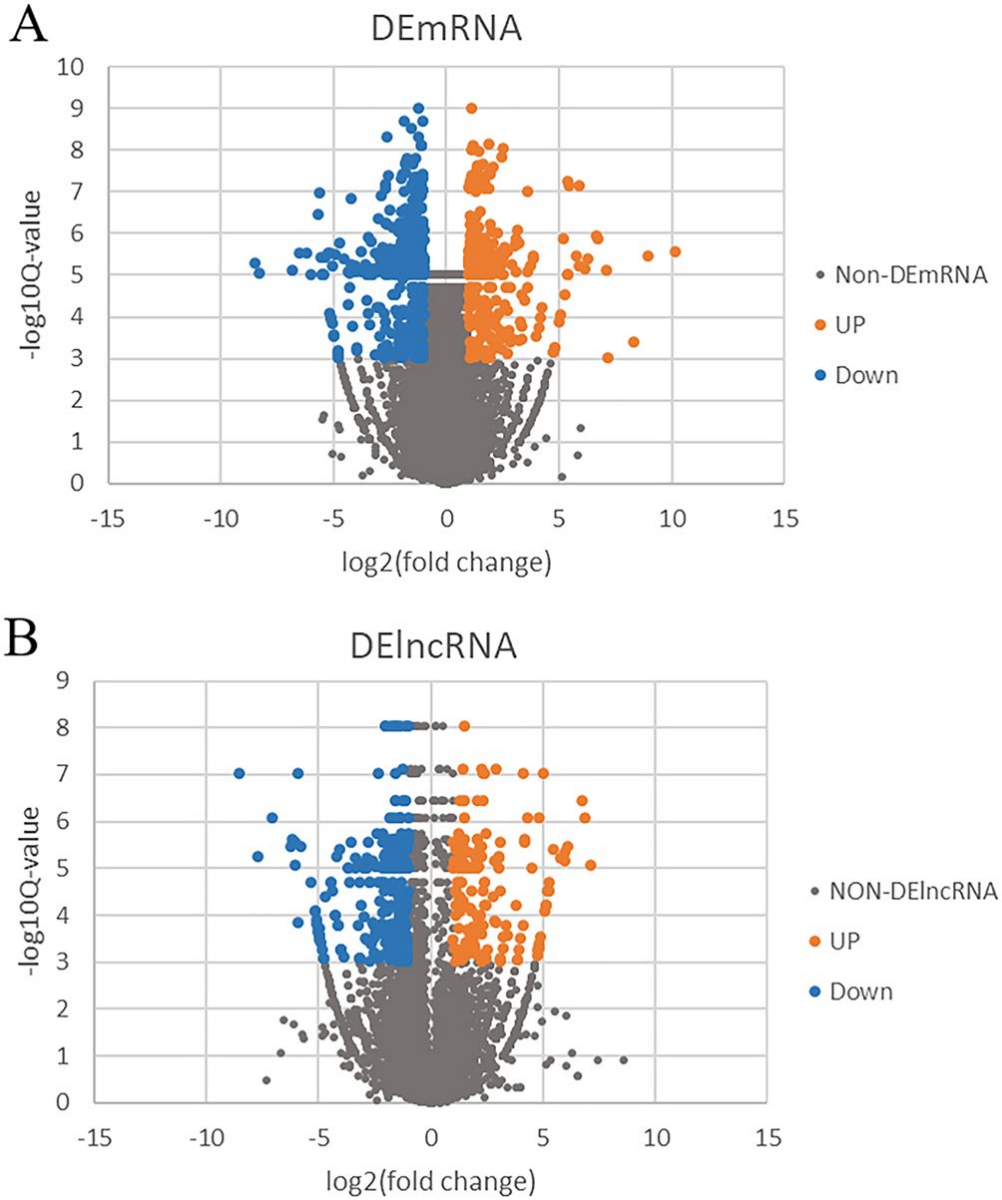
Different mRNAs expression amd different lncRNAs expression. (**A**) Volcano map of significantly different expression of mRNAs. Orange spots represent up-regulated mRNAs, and blue spots represent down-regulated mRNAs. (**B**) Volcano map of significantly different expression of lncRNAs. Orange spots represent up-regulated lncRNAs, and blue spots represent down-regulated lncRNAs.

To identify lncRNAs and mRNAs expression patterns that characterize CAD, we used the RNA-seq method. We found that 844 EAT-specific mRNAs (555 downregulated and 289 upregulated) and 395 lncRNAs (247 downregulated and 148 upregulated) were significantly different (CTRL vs. CAD; Cut-off: *p* < 0.001, |fold change| ≥ 2). The differentially expressed mRNAs (DEmRNAs) and differentially expressed lncRNAs (DElncRNAs) were shown in Table 3. To show the distribution of all differentially expressed RNA in the dimensions of -log (FDR) and logFC, we used the volcano plot (Fig. 1).

**Table 3:** The 20 most significantly upregulated and downregulated mRNAs and lncRNAs.

Upregulated mRNAs		Downregulated mRNAs		Upregulated lncRNAs		Downregulated lncRNAs	
mRNA	log2FC	mRNA	log2FC	lncRNA	log2FC	lncRNA	log2FC
SULT1A3	8.96584	PGBD3	− 6.82483	H19	10.14734	LINC00294	− 6.26844
ONECUT1	7.16101	RFLNA	− 6.19923	LINC02227	5.25198	IDS2	− 6.14591
XGY2	5.88797	LILRA3	− 5.97684	HOXB− AS3	4.63271	RALGAPA1P1	− 5.88859
SYT13	5.78803	CIB4	− 5.7138	HCG26	4.61375	LINC00311	− 4.97221
RGP1	5.40643	CATSPER4	− 5.59351	GOLGA8DP	4.45236	FGF14-IT1	− 4.94179
PHKG2	5.40579	RNASE2	− 5.52938	LINC02203	4.2563	MGC27345	− 4.92992
HSFX2	5.40012	KLRC4	− 5.5261	NOS2P3	4.15863	SMIM10L2B-AS1	− 4.91293
IL11	5.01752	CNTF	− 5.48205	LINC00994	4.09998	ACTA2-AS1	− 4.69639
TCP10L	4.49443	CHIA	− 5.22645	KIAA0087	3.73638	LINC00950	− 4.48137
ERVH48-1	4.45737	BPIFB4	− 5.14676	LINC01704	3.63049	MIR4422HG	− 4.39188
KCNA10	4.38948	ECEL1	− 5.01806	ABCC13	3.09998	LINC00601	− 4.39188
C7orf71	4.33605	MYH6	− 4.97684	LINC00906	2.85477	OR2L1P	− 3.97684
GOLGA6D	4.26204	PCDHA8	− 4.79787	UBE2E2-AS1	2.66702	BAALC-AS1	− 3.89438
INSM1	4.25198	ABCF1-DT	− 4.77039	LINC01781	2.48464	LINC01483	− 3.52938
HOXB5	4.21841	DHRSX	− 4.74735	LINC01778	2.45362	TSPEAR-AS2	− 2.8773
OR7D2	4.17798	KRT1	− 4.65491	LINC02242	2.37963	GAS5-AS1	− 2.86624
CNDP1	4.09597	SLC44A4	− 4.61838	LINC00473	2.25198	SNHG4	− 2.25678
SYCE3	4.01752	MROH3P	− 4.52938	GOLGA8EP	2.18518	LINC02518	− 2.18542
LGALS7	3.88425	PATE1	− 4.52938	DANT1	2.11363	LINC01352	− 2.16632
CYP1A1	3.84432	KCNRG	− 4.43872	LINC00235	2.01032	PVRIG2P	− 2.01901

*FC* fold change, *lncRNA* long noncoding RNA, *mRNA* messenger RNA.

### GO and pathway analysis of differentially expressed genes (DEGs)

**Figure 2:**
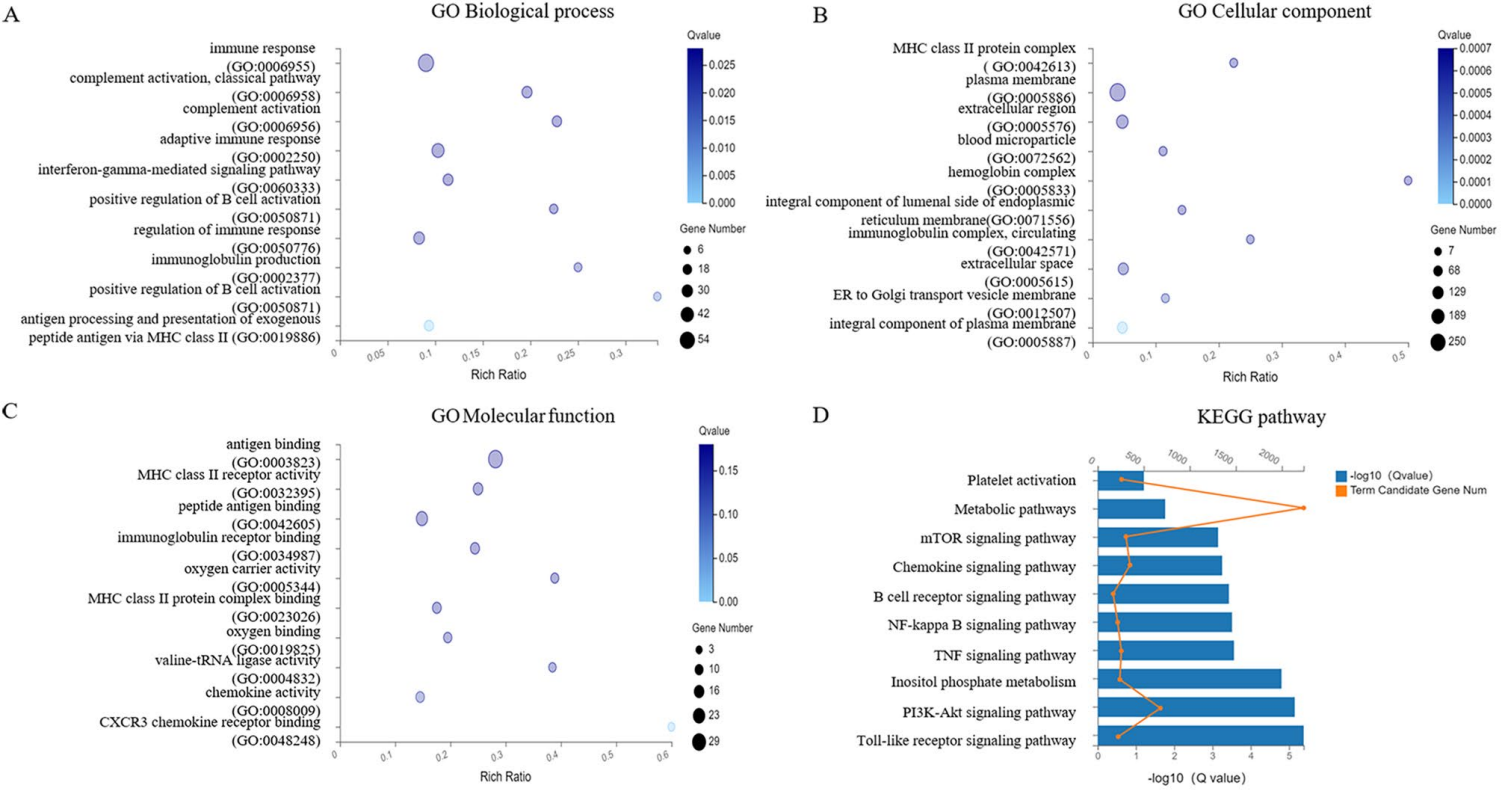
GO and KEGG Pathway enrichment analysis of differentially expressed long noncoding RNAs. (**A**–**C**) The y-axis represents the pathway entry, and the x-axis represents the rich ratio. The size of dot is directly proportional to the number of differentially expressed genes. The colors of each dots represent different Q values. (**A**) The biological process in which the target genes are significantly enriched; (**B**) Cell components in which the target genes are notably enriched; (**C**) molecular functions in which the target genes are markedly enriched. (**D**) KEGG pathways significantly associated with the target genes.

To provide a framework for the interpretation of our results, we then functionally clustered significant biological pathways by using GO and KEGG pathway analysis^12^. GO analysis showed changes in the biological processes (BP) of DEGs were enriched in the extracellular structure organization, collagen metabolic process, etc., as was shown in Fig. 2A. The changes in the cell component (CC) of DEGs were enriched in the extracellular matrix, apical part of the cell, contractile fibers, basal lamina, etc., as was shown in Fig. 2B. Figure 2C showed that the changes in molecular functions (MF) of DEGs were manifested in the functions of actin binding, platelet-derived growth factor binding, etc. Figure 2D showed the results of KEGG pathway analysis.

### Construction and analysis of the LINC00968-related ceRNA network

**Figure 3:**
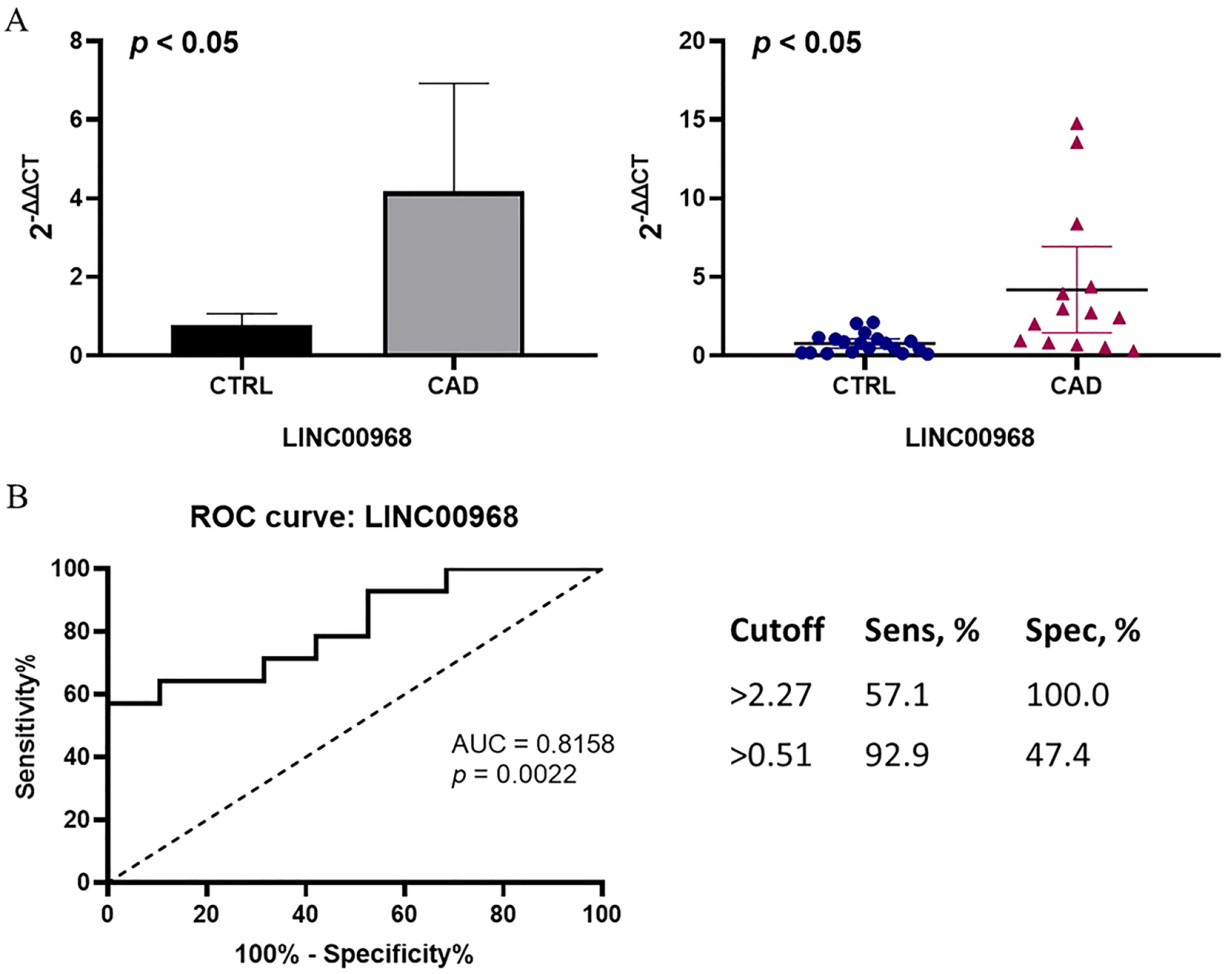
In CAD group, EAT overexpresses LINC00968; LINC00968 may act as a candidate biomarker of CAD. (**A**) Comparison of LINC00968 expression between CAD group and control group by verification via RT-qPCR. (**B**) ROC curve of LINC00968. The areas under the curve of LINC00968 was 0.8158 (*p* = 0.0022, 95% confidence interval 0.6653 to 0.9663).

**Figure 4:**
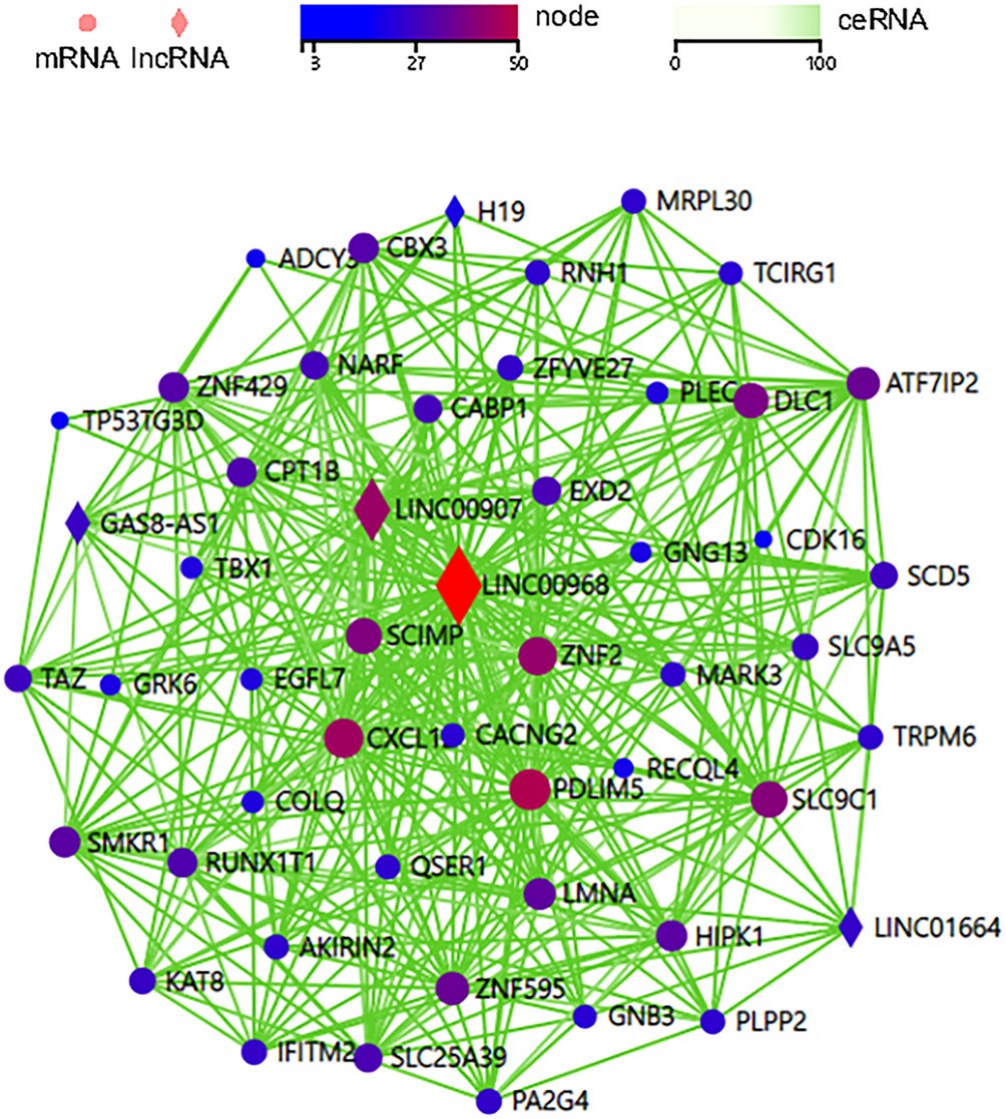
The Competing endogenous RNA network of LINC00968. The diamonds represent lncRNAs, and the circles represent mRNAs. From dark blue to deep red indicates more nodes between lncRNA-mRNA. The green line indicates an endogenous competitive relationship.

LncRNAs can act as ceRNAs to interfere with the function of specific miRNAs, thereby releasing the mRNAs affected by the miRNAs which can be predicted by softwares such as miRanda etc. We built the ceRNA network on the basis of the lncRNA and mRNA expression profiles in patients with CAD. Among those DElncRNAs, 28 lncRNAs were identified as lincRNAs and we found that LINC00968 level was more than 3-fold greater in CAD patients confirmed by RT-qPCR (Fig. 3A). Through logistic analyses, we found that LINC00968 level was an independent predictor of CAD when adjusted for possible confounding factors including sex, systolic blood pressure, hypertension and hyperlipidemia (Table 4). Furthermore, the ROC curve was built to assess the sensitivity and specificity of these lincRNAs as predictors for CAD, and the areas under the curve (AUC) were 0.8158 (*p* < 0.05) (Fig. 3B). ROC curve of LINC00968 relative expression with a cut off value of 2.27 achieved a sensitivity of 57.1%, and specificity of 100.0%. Then, we selected LINC00968 to build the network. The LINC00968-related ceRNA network was predicted based on the potential number of MREs shared by mRNAs and LINC00968, which includes 49 mRNAs. The network was shown in Fig. 4.

**Table 4:** Logistic regression analyses for the independent predictors of CAD.

	Univariate		Multivariate^a^	
	OR (95% CI)	*P* values	OR (95% CI)	*p* values
LINC00968	3.512 (1.289–9.571)	0.014	5.536 (1.242–24.671)	0.025

*OR* odds ratio, *CI* confidence interval.

^a^Adjusted for sex, SBP, hypertension and hyperlipidemia.

### PPI network construction and key mRNAs verification

**Figure 5:**
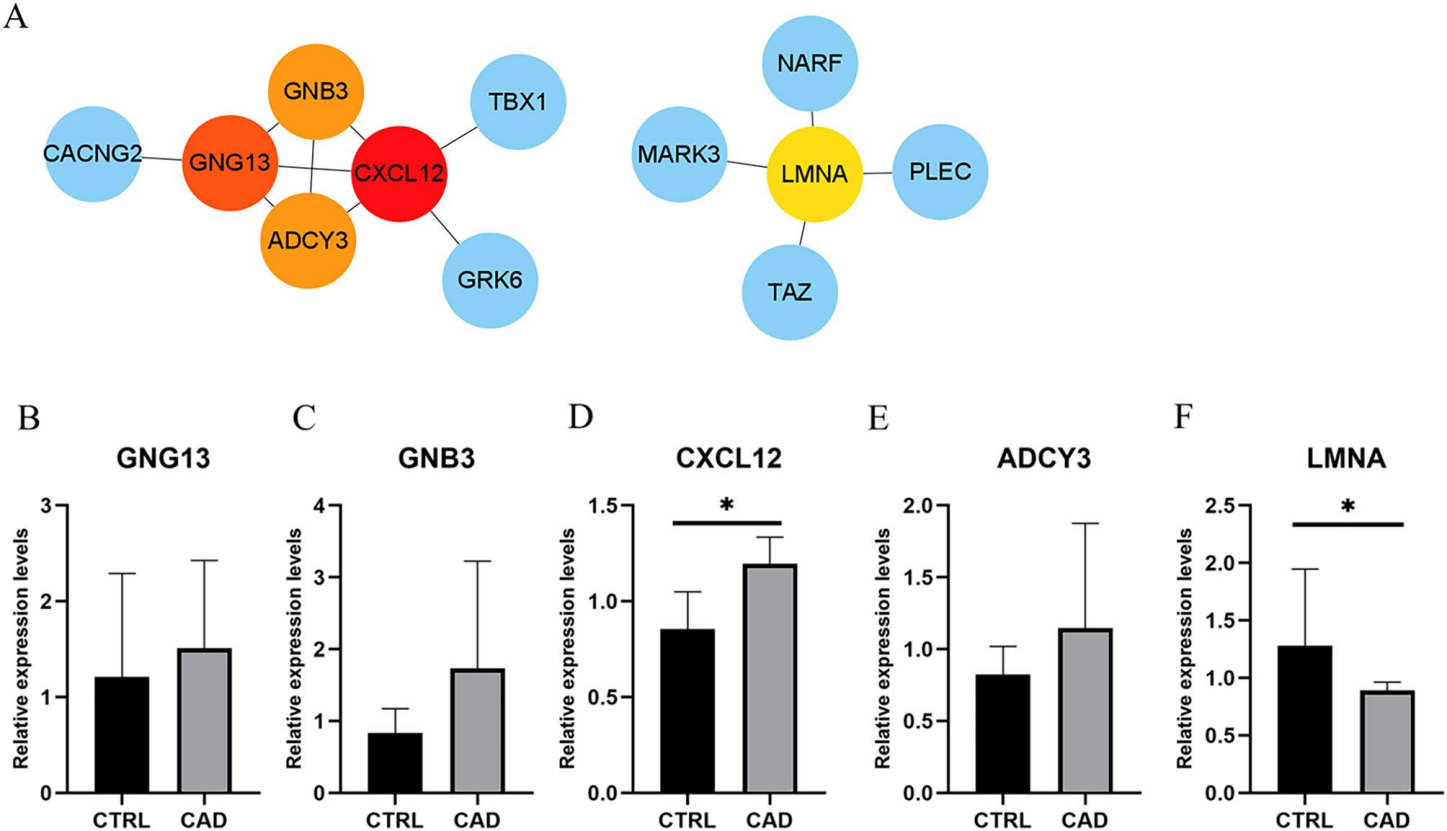
Analysis of PPI network and verification of top 5 key mRNAs in LINC00968-related ceRNA network. (**A**) The edges represented protein–protein associations which were meant to be specific and significant, the colored nodes (red and orange) represented top 5 key mRNAs identified by CytoHubba in Cytoscape. (**B**–**F**) qRT-PCR verification of top 5 key mRNAs in LINC00968 related ceRNA network. CXCL12 expression levels were significantly increased in EAT of CAD. Controversially, LMNA expression level was decreased in EAT of CAD. *p < 0.05. In each column, error bars show the mean and standard deviation per group.

To further identify key mRNAs in the LINC00968-related ceRNA network, mRNAs of the ceRNA network were submitted to the STRING database. PPI network was constructed with a comprehensive score greater than 0.4 (Fig. 5A), and the most important module was obtained using MCODE in Cytoscape. The key mRNAs were selected from the PPI network using the maximal clique centrality (MCC) algorithm of CytoHubba. The top 5 key mRNAs identified by MCC were GNG13, GNB3, CXCL12, ADCY3 and LMNA. RT-qPCR was performed using the total RNA extracted from EAT to verify the expression levels of key mRNAs. The results of RT-qPCR showed significant changes in the expression levels of key mRNAs such as CXCL12 (0.855 ± 0.194 vs. 1.197 ± 0.138, CTRL vs. CAD, *p* = 0.021) and LMNA (1.197 ± 0.138 vs. 0.890 ± 0.072, CTRL vs. CAD, *p* = 0.047) in Fig. 5.

## Discussion

EAT is a type of visceral adipose tissue located between the myocardium and the pericardium. The lineage tracing study confirms that EAT is differentiated from epicardial cells^13^. In addition to acting as a buffer zone around the heart, EAT also acts as an energy reservoir for myocardial tissue to provide free fatty acids for cardiomyocyte metabolism^14^. Under pathological condition, EAT exhibits as metabolically active endocrine and paracrine organ that secretes a large amount of anti-inflammatory and pro-inflammatory adipokines, affecting the dynamic balance of glycolipid metabolism, cardiac structure and function^15–17^. EAT is directly nourished by coronary arteries, and there is no fascia isolation between EAT and myocardial tissue, which provides an important anatomical basis for the interaction among them^18^. Several evidence-based medical studies have confirmed that EAT is closely related to the occurrence and development of CAD^19,20^, and may be associated with the increase in the incidence of long-term adverse events.

However, thus far, most of these studies are observational. Mechanism research and prospective studies are sparse. It is, therefore, necessary to understand whether changes in EAT physiology are the cause of atherosclerosis or just an epiphenomenon of metabolic syndrome. Here, we provide novel information that describes the molecular identity (mRNAs and lncRNAs) of EAT in CAD and non-CAD by performing a comprehensive analysis of the expression profiles. Through GO and KEGG analyses, we further analyzed the pathways and functions in which the DEGs are involved. We present evidence that, compared with CTRL, EAT of CAD shows enhanced inflammation and promotion of lipid metabolic disorder.

Intergenic lncRNAs (lincRNAs) are defined as non-coding RNAs longer than 200 nucleotides without overlapping protein-coding genes, which are distinguished from the general lncRNA transcripts, as a lot of lncRNAs share sequence with coding loci^21^. LincRNAs are important regulators of gene expression and often exhibit remarkable tissue-specificity^22^. One of the potential regulative mechanisms of lincRNAs is to act as ceRNAs. miRNAs can bind to mRNAs through MREs, causing mRNAs degradation or inhibiting its translation. When lincRNAs and mRNAs share the same MREs, they form a relationship of competition for the same type of miRNAs, thus preventing mRNAs degradation, and in such manner lincRNA indirectly regulates the expression level of mRNA^23,24^. The ceRNA mechanism shows a novel way of studying lncRNA-mRNA-miRNA cross talk and has received wide attention. Some studies have showed and highlighted the core function of ceRNA network across CVD. lncRNA HIF1A-AS1 acts as a ceRNA of miR-204 and elevates SOCS2 expression, which contributes to ventricular remodeling after myocardial ischemia/reperfusion injury^25^. Another research by Liang et al.^26^ identified the lncRNA 2810403D21Rik/Mirf can act as a ceRNA of miRNA26a in ischemic myocardial injury. The lncRNA TNK-AS1 regulates smooth muscle cell proliferation and migration via acting as a ceRNA of miR-150-5p^27^. The uncovering of the ceRNA mechanism may provide a new layer for exploring the physiopathology process and shed new light on cardiovascular research.

In this study, we observed an adipose tissue-specific lincRNA of high expression, namely LINC00968, which appeared to be significantly dysregulated in CAD. The ROC analysis suggests that LINC00968 may be used as a potential target for further study. Although LINC00968 was previously reported to be connected to the function of endothelial cells^28^, the mechanism of its increase in an adipose tissue-specific manner is still unclear. Here, we applied the bioinformatics method to construct the LINC00968-related ceRNA regulatory network, which provides new ideas for exploring the biological functions of LINC00968. From the constructed LIN00968 related ceRNA network, we found several mRNAs which have been previously reported to play a role in atherosclerosis, such as CXCL12^29^, TAZ^30^, PDLIM5^31^, GNB3^32^, LMNA^33^, TRPM6^34^. Similarly, we used bioinformatics to predict the top five key mRNAs in the LINC00968-related ceRNA network, including GNG13, GNB3, CXCL12, ADCY3 and LMNA, among which CXCL12 and LMNA were significantly dysregulated in CAD according to verification results. LINC00968 may interact with specific miRNAs and cause the dysregulation of those genes. Interestingly, in the network, we found that lncRNA H19 was significantly down regulated, while LncRNA H19 is a significant CAD-associated lncRNA^35^. These results might help us to study EAT’s role in CAD from a new perspective. In the future, we will conduct an in-depth study of the regulatory mechanisms underlying the LINC00968 ceRNA network.

## Limitations

The main limitation of the study could be the limited amount of total adipose tissue obtained from human due to ethical restrictions. In addition, the present study stopped short of validating the functions of LINC00968 and further exploring the related mechanisms because of the limitations in methodology of culturing human epicardial adipose tissue cells. Further studies are required to gain insight into the pathogenesis.

## Conclusion

In conclusion, our study has identified expression profiles of lncRNAs in EAT that potentially predict the progression of CAD by using RNA-seq. Among them, LINC00968 was identified and its related ceRNA network may play a pivotal role in the process of CAD pathophysiology.

## Materials and methods

### Patients, samples, and ethics

43 non-diabetic caucasian patients were recruited at Xiangya Hospital of Central South University; 24 underwent cardiac valve surgery (CTRL group; no evidence of CAD), and 19 underwent coronary artery bypass graft surgery (CAD group). Inclusion criteria of CAD group: CAD patients undergoing coronary artery bypass surgery (confirmed by coronary angiography); inclusion criteria of CTRL group: patients undergoing valve replacement for valvular diseases or surgeries for congenital heart diseases, with age, gender, and BMI matched with CAD group and with no evidence of coronary heart disease or negative findings in coronary angiography. Exclusion criteria include history of PCI, history of thoracotomy, diabetes mellitus, tumor, severe infection, renal insufficiency (glomerular filtration rate < 60 mL/min), active period of autoimmune disease, and severe obesity BMI > 35 kg/m^2^. Specimens were collected in the way as previously described with informed consent signed^36^. EAT samples were collected from the atrioventricular groove near the right coronary artery. EAT samples were immediately snap-frozen and stored using liquid nitrogen for RNA-seq and qRT-PCR analysis. The research design was approved by the Medical Ethics Committee of the Xiangya Hospital of Central South University.

Hypertension was defined as: systolic pressure ≥ 140 mmHg or diastolic pressure ≥ 90 mmHg without the use of antihypertensive drugs, or blood pressure within the normal range with self-reported use of antihypertensive drugs. Diabetes mellitus was identified if the random plasma glucose concentration was ≥ 11.1 mmol/L, or the fasting plasma glucose concentration was ≥ 7.0 mmol/L, or the postprandial plasma glucose concentration (2 h after meals) was ≥ 11.1 mmol/L in oral glucose tolerance test. Hyperlipidemia was diagnosed if the total cholesterol was ≥ 6.22 mmol/L (240 mg/dL), and/or triglyceride was ≥ 2.26 mmol/L (200 mg/dL), and/or low-density lipoprotein cholesterol was ≥ 4.14 mmol/L (160 mmol/L).

### Total RNA extraction and lncRNA library construction

Total RNA was extracted from EAT samples using TRIzol (Invitrogen, Carlsbad, CA, USA) according to the manufacturer's instructions. The RNA quality and quantity were evaluated using NanoDrop 2000 spectrophotometer (Thermo Fisher Scientific, MA, USA) and Agilent 2100 bioanalyzer (Thermo Fisher Scientific, MA, USA).

The Ribo-Zero Magnetic Kit (Epicentre) was used to process approximately 1 μg of total RNA in each sample to deplete rRNA. The First Strand Master Mix (Invitrogen) was added to fragment the retrieved RNA. The synthesized cDNA was end-repaired and then 3′adenylated. The ends of these 3′adenylated cDNA fragments were connected using adaptors. PCR Primer Cocktail and PCR Master Mix were used for PCR amplification to enrich cDNA fragments. The PCR product was then purified using Ampure XP beads. Two methods were used for the qualification and quantification of the final library: using Agilent 2100 bioanalyzer to check the size distribution of fragments, and using real-time quantitative PCR (RT-qPCR) (TaqMan Probe) for the library quantification. Qualified libraries were sequenced on the Hiseq 4000 or Hiseq X-ten platform (BGI, Shenzhen, China).

### Prediction of lncRNA target genes

To identify lncRNA cis-regulated target genes, we screened for protein-coding genes located within 10 kb upstream or 20 kb downstream of a differentially expressed lncRNA. The Pearson and Spearman correlation coefficient was calculated. To identify an mRNA as a lncRNA trans-regulated target gene, the correlation coefficient between a lncRNA and mRNA pair r ≥ 0.6.

### Gene ontology (GO) and pathway analysis

Quantitative signal data for each gene from CAD and control EAT samples were compared to obtain absolute fold change (FC) values, which were then log2 FC transformed. Differential expression of mRNAs and lncRNAs was defined by a |log2 FC| ≥ 1 and Q value < 0.05. To predict the potential biological roles of the identified lncRNAs, we performed enrichment analyses on differentially expressed mRNAs and lncRNAs targeted genes using GOseq and KOBAS (2.0), respectively. Those enriched GO terms or pathways with a Q value < 0.05 were considered significant.

### RT-qPCR of differentially expressed lncRNAs

RT-qPCR was performed to validate the lncRNAs expressions identified by the RNA-seq. We selected differential intergenic lncRNAs (lincRNAs) with the highest absolute expression levels and the largest FC between the CAD and control groups from RNA-seq for RT-qPCR validation. LINC00968 forward primer, 5′-AAGCTGAAAAGCTGCCTGGT-3′; LINC00968 reverse primer, 5′-TGGGAGCCGATTCTCCAAGG-3′; Beta-actin forward primer, 5′-TGGACTTCGAGCAAG AGATG-3′; Beta-actin reverse primer, 5′-TGTTGGCGTACAGGTCTTTG-3′. Complementary DNAs (cDNAs) were synthesized from the total RNA using Mir-X miRNA First-Strand Synthesis Kit (cleantech, USA). The selected lincRNAs were quantified by performing RT-qPCR on an ABI 7500 real-time PCR system with SYBR Premix Ex Taq RT-qPCR assays (TaKaRa, Japan). Beta-actin was used as an endogenous reference for the normalization of all reactions. The method of 2-ΔΔCt was used to compare the expression levels between the two groups.

### ceRNA network analysis

The ceRNA network was based on the ceRNA hypothesis^11^ that ceRNAs could co-regulate each other by competing for shared MREs. To construct the network, lncRNA/mRNA-miRNA interactions were retrieved from miRDB (http://mirdb.org/), miRcode (http://www.mircode.org/), miRTarBase (http://mirtarbase.mbc.nctu.edu.tw/). Then, differentially expressed lncRNA and mRNA with FDR < 1%, |log2 FC| ≥ 1 and Q value < 0.05 were retained to establish the ceRNA network. Dr. Tom's network platform of BGI (https://biosys.bgi.com) was applied to construct and visualize the network.

### PPI network construction and analysis

A search tool (STRING 10.5; http://string-db.org)^37^ was used to establish a PPI network, and Cytoscape^38^ was used to draw the network. The most important modules in the PPI network were identified using MCODE in Cytoscape, with threshold as: MCODE scores > 5, degree cut-off = 2, node score cut-off = 0.2, max depth = 100 and k-score = 2. The MCC algorithm of CytoHubba was used to explore the key genes of the PPI network.

### Statistical analysis

The measurement data were expressed as mean ± standard deviation, and the independent sample *t* test was used to compare the data groups that met the normal distribution. For those that did not conform to the normal distribution, the Mann–Whitney U test was used. The enumeration data were shown as a percentage, and the chi-square test was used for comparison between groups. *p* values obtained from each gene via the Wald test were converted by multiple test correction into Q values. The expression levels of lncRNA were normalized to endogenous reference (beta-actin). Differential expression between the CAD and control groups was expressed as the median and the interquartile range. Univariate and multivariate logistic regression analysis were performed to determine the independent predictor of CAD. The receiver operating characteristic curve (ROC curve) was used to evaluate the predictive value of differentially expressed lncRNA for CAD. The statistical software SPSS 25.0 was utilized for nonparametric Mann Whitney U tests of the real-time PCR results. Two-tailed *p* < 0.05 indicates a statistically significant difference.

### Ethics statement

We confirm that all methods were carried out in accordance with relevant guidelines and regulations.
